# The polymorphic landscape analysis of GATA1 exons uncovered the genetic variants associated with higher thrombocytopenia in dengue patients

**DOI:** 10.1371/journal.pntd.0010537

**Published:** 2022-06-30

**Authors:** Razoan Al Rimon, Mohammad Sayem, Saruar Alam, Abdullah Al Saba, Mousumi Sanyal, Md. Robed Amin, Ahmedul Kabir, Sajib Chakraborty, A. H. M. Nurun Nabi

**Affiliations:** 1 Laboratory of Population Genetics, Department of Biochemistry and Molecular Biology, University of Dhaka, Dhaka, Bangladesh; 2 Translational Systems Biology Laboratory, Department of Biochemistry and Molecular Biology, University of Dhaka, Dhaka, Bangladesh; 3 Department of Medicine, Dhaka Medical College, Dhaka, Bangladesh; Minia University, EGYPT

## Abstract

The current study elucidated an association between gene variants and thrombocytopenia through the investigation of the exonic polymorphic landscape of hematopoietic transcription factor—GATA1 gene in dengue patients. A total of 115 unrelated dengue patients with dengue fever (DF) (N = 91) and dengue hemorrhagic fever (DHF) (N = 24) were included in the study. All dengue patients were confirmed through detection of NS1 antigen, IgM, and IgG antibodies against the dengue virus. Polymerase chain reaction using specific primers amplified the exonic regions of GATA1 while Sanger sequencing and chromatogram analyses facilitated the identification of variants. Variants G>A (at chX: 48792009) and C>A (at chX: 4879118) had higher frequency out of 13 variants identified (3 annotated and 10 newly recognized). Patients carrying either nonsynonymous or synonymous variants had significantly lower mean values of platelets compared to those harboring the reference nucleotides (NC_000023.11). Further analyses revealed that the change in amino acid residue leads to the altered three-dimensional structure followed by interaction with neighboring residues. Increased stability of the protein due to substitution of serine by asparagine (S129N at chX: 48792009) may cause increased rigidity followed by reduced structural flexibility which may ultimately disturb the dimerization (an important prerequisite for GATA1 to perform its biological activity) process of the GATA1 protein. This, in turn, may affect the function of GATA1 followed by impaired production of mature platelets which may be reflected by the lower platelet counts in individuals with such variation. In summary, we have identified new variants within the GATA1 gene which were found to be clinically relevant to the outcome of dengue patients and thus, have the potential as candidate biomarkers for the determination of severity and prognosis of thrombocytopenia caused by dengue virus. However, further validation of this study in a large number of dengue patients is warranted.

**Trial Registration:** number SLCTR/2019/037.

## Author summary

Dengue, a mosquito-borne viral disease, caused by dengue virus (DENV) of the Flaviviridae family has been the source of a global epidemic for decades, and it has recently spread to all parts of the globe. Hemorrhagic manifestations in dengue are caused by increased vascular permeability and thrombocytopenia (150K cells/L). GATA1 is considered to be the master transcription factor for regulating the differentiation and maturation of megakaryocytes (MKs). The exonic polymorphism landscape of hematopoietic transcription factor—GATA1 in dengue patients was investigated in our study to see if there is an association between genetic variations and thrombocytopenia. Out of the 13 variations identified, variants G>A (chX:48792009) and C>A (chX:4879118) had the highest frequencies. Patients harboring either nonsynonymous or synonymous variants had significantly lower mean platelet counts compared to those having wild-type nucleotides. We have identified new GATA1 gene variations that were proven to be clinically related to the outcome of dengue patients and hence holds the potential to be candidate biomarkers for determining the severity and prognosis of dengue thrombocytopenia. However, this study needs to be replicated in a large number of dengue patients.

## Introduction

Dengue, the mosquito-transmitted viral disease, caused by dengue virus (DENV) has been the reason for the global pandemic for decades, and recently it has spread across all regions of the world. Approximately 390 million people are at greater risk of getting dengue infection each year [1]. Clinical dengue fever (DF) progresses with a sudden onset of high fever after an incubation period of 3–15 days (normally 5–8 days) [1,2]. However according to World Health Organization (WHO), nearly half of the dengue virus infected patients may develop dengue hemorrhagic fever (DHF) with plasma leakage [3]. Increased vascular permeability and thrombocytopenia (< 150K cells/μL) are the causal factors of these hemorrhagic manifestations [4]. Although platelet count ranging between 30,000 to 50,000 cells/μL, occasionally manifests as purpura, platelet count less than 30,000 cells/μL may cause bleeding with minimal trauma [5]. Subsequently, when the platelet level drops below 5,000 cells/μL, severe outcome of dengue infections occurs with spontaneous bleeding [6]. In 2009, WHO recommended that a significant drop in platelet count or a current platelet count of less than 150,000/mm^3^ is one of the markers of waning dengue infection [7].

Thrombocytopenia develops as a result of platelet dysfunction, which includes increased platelet activation [8], clot formation [9], apoptosis [10], and inflammatory cytokine production [10–12]. However, understanding DENV pathogenesis remains a difficult task, as further studies are required to fully comprehend the virus’s complex interactions with its host. It is proven that several risk factors including host genetic background [13] can modulate the severity of DENV infection. As a result, variation in host genetics particularly in the genes involved with thrombopoiesis may potentiate the complications associated with dengue infection.

In normal physiological process, the subsequent differentiation of hematopoietic stem cells leads to the production of the megakaryocytes (MKs) which further develops to generate mature platelets and the differentiation and maturation of MKs are regulated by several transcription factors [14,15]. Among the transcription factors, GATA1 is considered to be the master transcription factor [16]. GATA1 consists of two different untranslated first exons, IT and IE, and five translated exons, II to VI [4,17]. It is located in X-chromosome (Xp11.23). The mature protein comprises of two zinc finger domains and one activation domain [18,19]. The role of GATA1 protein in megakaryocytes is to recruit transcriptional cofactors, such as FOG (friend of GATA1) to megakaryocyte-expressed genes like NFE2. NFE2 acts as a regulator of proplatelet formation by promoting the final stage of maturation of MKs [20]. Similarly, GATA1 cooperates with Fli-1 for the activation of genes like CD41, CD42b, and GPIX which are associated with the terminal differentiation of megakaryocytes [21]. Substantial studies have documented that mutations in the GATA1 gene results in X-linked thrombocytopenia with thalassemia and X-linked thrombocytopenia with dyserythropoietic anemia. As a result, GATA1-mutated patients have decreased number of platelets with varying degrees of anemia and irregular red blood cell morphology [22].

Infections caused by dengue virus, hepatitis B virus, hepatitis C virus as well as diseases like Down syndrome are associated with thrombocytopenia. Genetic variants of certain GATA1 gene can lead these people to a state of higher risk and thus, preclinical analysis can help to take necessary steps before the impact of the diseases occurs. So, the objectives of this study are to (i) analyze entire exonic regions of the GATA1 gene in dengue patients suffering from thrombocytopenia, (ii) identify the relationship between different variants of the GATA1 gene with thrombocytopenia, and (iii) elucidate the impact of variants on the three-dimensional structure of GATA1 followed by its function. This study will facilitate finding out the underlying genetic cause of thrombocytopenia related to dengue virus infection with respect to host genetics which may ultimately contribute to early prediction of patients with higher risk of thrombocytopenia in terms of disease progression and prognosis.

## Materials and methods

### Ethics statement

This study is an extra work of a Phase II Clinical Trial, which was approved by the ethical review committee of the Bangladesh Medical Research Council (ID: BMRC/NREC/2019/171) and registered with the international clinical trial registry, number SLCTR/2019/037 (https://slctr.lk/trials/slctr-2019-037). Written consent from each participant was obtained prior to blood collection.

### Study design

A total of 115 patients were recruited from Dhaka Medical College Hospital, Dhaka; AMZ Hospital, Dhaka and Better Life Hospital, Dhaka as part of the clinical trial (international clinical trial registry number: SLCTR/2019/037) [23]. Patients who demonstrated clinical manifestations of dengue virus infection like leukopenia, increased hematocrit, reduced platelet count, headache, bone pain, rash were suspected as dengue patients according to the guideline of the World Health Organization [24]. They were confirmed through the positive results of dengue specific antigen (NS1) and antibody (IgM/IgG) tests. Both viral antigens NS1 as well as IgM and IgG antibodies were detected by commercially available kits that used enzyme-linked immunosorbent assay (ELISA) as described in our recently completed study [23]. Pregnant women, patients with thrombocytopenia (due to causes other than dengue), AST/ALT level greater than 5 times of the normal upper limit, past portal vein thrombosis, infection with HCV,HVB or having chronic liver disease, history of taking immunosuppressive therapy and severe co-morbidity were excluded from the study (https://slctr.lk/trials/slctr-2019-037).

**Fig 1:**
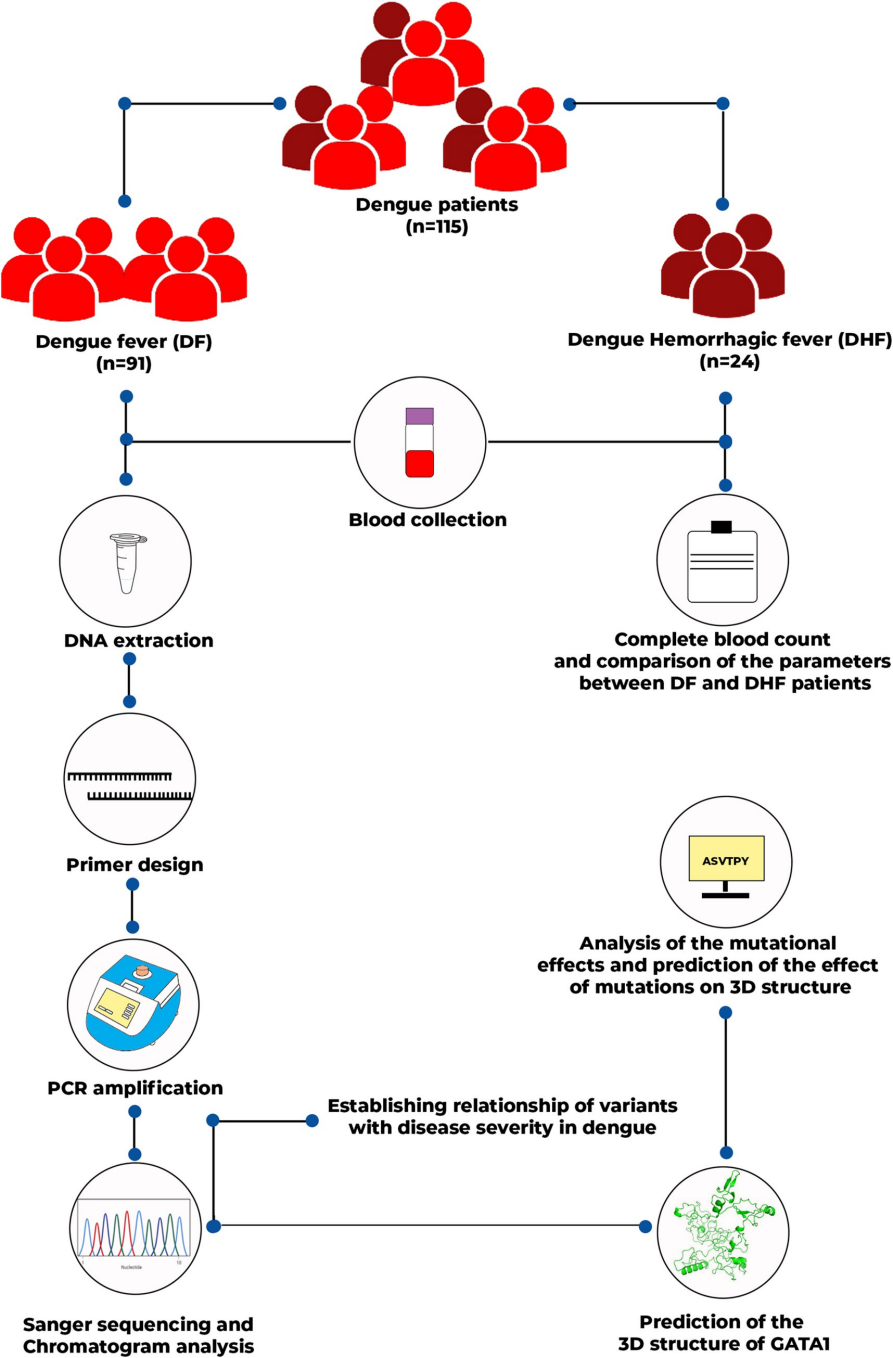
Graphical representation of the methodology undertaken during the study.

The patients were then grouped into two classes: dengue fever (DF) and dengue hemorrhagic fever (DHF) according to the suggestion of the World Health Organization as described in our recent work [24]. Patients were considered to have moderate dengue who exhibited high fever (40°C/104°F) along with two of the cautionary signs including abdominal pain and tenderness, persistent vomiting, clinical fluid accumulation, mucosal bleeding, lethargy, restlessness, and liver enlargement, increase in hematocrit followed by quick reduction of platelet count. On the other hand, patients who had one of the following symptoms: plasma leakage leading to shock or respiratory distress, severe bleeding, or organ failure (eg, elevated liver enzyme levels, impaired consciousness, or heart failure) were grouped into DHF [25,26]. The outline of the methodology of the study is shown in Fig 1.

### Blood collection and hematological analyses

After getting written consent from each participant, five milliliters of blood was collected in an EDTA containing vacutainer tube with the help of expert phlebotomists. Blood was transferred to the laboratory of population genetics using a sample carrier. Complete Blood Count (CBC) analyses were performed using the Sysmex XN-2000 Hematology Analyzer that uses the direct current sheath flow detection method. From CBC analyses RBC Count, hematocrit level, mean corpuscular volume (MCV), mean corpuscular hemoglobin (MCH), mean corpuscular hemoglobin concentration (MCHC), RBC distribution width (RDW), total WBC count, differential count including percentages of neutrophils, lymphocytes, monocytes, eosinophils, basophils and eosinophils were determined.

### DNA extraction, polymerase chain reaction and determination of entire coding sequence of hematopoietic transcription factor GATA1

Genomic DNA was extracted from the cellular fraction of the collected blood, quality and quantity of the extracted DNA were evaluated according to our previous methods [27–29]. Primers to amplify exonic regions of GATA1 were designed using Primer3 web-based platform [30]. GATA1 gene consists of 6 exonic regions. Prior to sequencing, a total of 4 primers covering 5 exons (from 2 to 6) were designed to amplify exonic regions (except exon 1 because it remains untranslated) [17,31]. The list of the primers including sizes of each amplicon has been shown in S1 Table. Each pair of primer was obtained from IDT, USA. After dilution of primers, polymerase chain reaction was optimized for each set of primer using the conditions as demonstrated in S2 Table.

### Sequencing of PCR amplicons and analyses of chromatograms

A total of 460 amplicons for four pairs of primer sets targeted to amplify the entire GATA1 exonic regions were subjected to Sanger sequencing. After analyzing the chromatograms, to increase the confidence of base call, total forty samples were resequenced using fresh PCR amplicons. The chromatograms of the amplicons were analyzed by aligning and comparing the results with the reference sequence of GATA1, NC_000023.11 that was retrieved from NCBI (www.ncbi.com). Sequences of the reference exonic regions containing known genetic variation [identified by SNPmasker 1.1 [32]] were also used to compare with the sequences. The alignment and analyses of the sequences were performed using Geneious Prime software version 2020.0.2 (https://www.geneious.com/)).

### Assessment of the functional impacts of nonsynonymous mutations

Functional effects of nonsynonymous mutations found within the exonic regions of GATA1 were analyzed using different web-based tools. First, SIFT was used to predict tolerated and deleterious substitutions for every position of the GATA1 nucleotide sequence [33]. Then, PolyPhen-2 was used to predict possible impact of an amino acid substitution on the structure and function of GATA1 protein [34]. After that, SNPs&GO was used to predict GATA1 point mutations that has the potential to become single handedly reasonable to cause disease in human [35]. Followed by this, PhD-SNP was utilized to classify GATA1 point mutations into disease related and neutral polymorphism class [36]. Subsequently, PANTHER-PSEP was used to estimate the likelihood of a particular nonsynonymous coding SNP to cause a functional impact on GATA1 protein [37]. Finally, I-Mutant 2.0 was used to analyse the protein stability and alterations by taking into account the single-site mutations on GATA1 protein structure [38].

### Prediction and validation of three-dimensional structures of GATA1 protein

At first, to validate the effects of the nonsynonymous SNPs on the structure and function of GATA1 protein, the 3D model of GATA1 protein was generated using RaptorX web server tool. The three-dimensional structures were visualized using the PyMOL Molecular Graphics System, Version 2.4.1 Schrödinger, LLC. The quality of the predicted three dimensional structure of GATA1 protein was validated first using PROCHECK. Ramachandran Plot of the modelled structure was generated for the assessment of the overall quality of the structure to be accepted for further use. After that, PROSA was used to check 3D models of GATA1 protein structures for potential errors. ERRAT2 was used to assess the quality of the structure by comparing with highly refined structures that is available in database. Followed by that, MISSENSE3D was used to observe the effect of a missense variant on GATA1 protein structure through different parameters including relative solvent accessibility (RSA), disulfide bond breakage, charge introduction, secondary structure alteration, H-bond breakage, cavity alteration etc. Finally, MODELLER was used for mutant protein structure modeling by using the initial modelled 3D structure as template and the structures were then compared by measuring distances between wild amino acids’ atoms and mutant amino acids’ atoms with nearby amino acids’ atoms [39].

### Statistical analyses

The results were expressed as mean±SEM (Standard Error of Mean) for continuous variables and as a percentage for categorical variables. To compare the differences between different variables obtained from the two groups of dengue patients with thrombocytopenia, data were analyzed using IBM SPSS Statistics for Windows, Version 23.0. Armonk, NY: IBM Corp. and GraphPad Prism version 8.0.0 for Windows, GraphPad Software, San Diego, California USA, www.graphpad.com. A p-value of less than 0.05 was considered statistically significant.

Association of platelet counts with the GATA1 exonic region variants was performed using R programming language. The differences between the mean platelet counts of the mutant heterozygous genotype and the wild type homozygous were calculated. The association was also adjusted for age and dengue fever type (DF and DHF). The number of study participants were stratified into five groups based on the number of mutations they harbored. Association of the platelet counts with these groups was conducted using R. The association was also adjusted for age and dengue fever type (DF and DHF).

## Results

Out of 115 dengue patients enrolled in this study, 80 were male (69.57%) and 35 were female (30.43%). All the patients were dengue infected and had thrombocytopenia on their first day of hospitalization. Detection of non-structural protein NS1 as well as determination of the levels of immunoglobulin G (IgG), and immunoglobulin M (IgM) were used to confirm dengue virus infection. Thrombocytopenia was confirmed using platelet count. According to the guidelines of the World Health Organization [23], 91 patients (79.13%) were suffering from dengue fever (DF) while 24 patients (20.87%) had dengue hemorrhagic fever (DHF) among the total participants. Table 1 represents the data of complete blood analyses of the study participants. The average age of the patients was 27.18±0.78 years, where the DHF occurs in relatively older (p = 0.001) people with a mean age of 32.21±2.21 years compared to DF whose average age was 25.86±0.75 years. All the patients in the study had thrombocytopenia, with the average platelet count 60.18±3.66 K cells/μL. The mean platelets of patients with DHF (48.58±6.86 K cells/μL) were lower than that of DF 63.24±4.21 K cells/μL though statistically insignificant. Among the study participants, DHF occurred in older patients compared to their DF counterparts. The comparison of other blood parameters between DHF and DF has been shown in Table 1.

**Table 1:** Comparison of blood parameters between dengue patients with dengue fever (DF) and dengue hemorrhagic fever (DHF).

Parameters	Reference Values	Total Dengue Patients (M ±SEM)	Stratified Dengue Patients (Mean ±SEM)		P DF vs DHF	Patients with DF (Mean ±SEM)		P ♂ vs ♀	Patients with DHF (Mean ±SEM)		P ♂ vs ♀
			DF (n = 91)	DHF (n = 24)		Male, ♂ (n = 65)	Female, ♀ (n = 26)		Male (n = 15)	Female (n = 9)	
Age (years)	-	27.18±0.78	25.86±0.75	32.21±2.21	0.001**	25.6±0.92	26.5±1.27	0.591	31.59±2.88	33.71±3.17	0.672
PLT (K cells/μL)	150–450	60.18±3.66	63.24±4.21	48.58±6.86	0.103	58.48±4.86	75.15±8.01	0.073	49.47±8.56	46.43±11.96	0.845
Hb (g/dL)	12–16	14.49±0.23	14.56±0.28	14.21±0.35	0.549	15.3±0.33	12.7±0.34	0.000**	14.84±0.38	12.7±0.36	0.003**
RBC (M cells/μL)	3.5–6.0	5.13±0.08	5.11±0.09	5.2±0.14	0.648	5.35±0.09	4.52±0.15	0.000**	5.44±0.15	4.62±0.15	0.004**
HCT (%)	37–52	41.86±0.49	41.9±0.57	41.7±0.98	0.868	43.67±0.56	37.5±1.01	0.000**	43.31±1.1	37.79±1.13	0.007**
MCH (pg)	27–32	28.25±0.26	28.46±0.3	27.44±0.45	0.109	28.52±0.39	28.32±0.4	0.769	27.4±0.58	27.53±0.68	0.900
MCHC (g/dL)	30–35	34.2±0.1	34.23±0.11	34.08±0.22	0.562	34.36±0.12	33.9±0.22	0.063	34.25±0.29	33.69±0.27	0.257
WBC (K cells/μL)	4.0–11.0	5.58±0.28	5.54±0.29	5.76±0.8	0.756	5.35±0.35	6.00±0.50	0.320	5.43±1.01	6.55±1.31	0.537
Neutrophil (%)	50–70	49.11±1.48	48.16±1.6	52.71±3.72	0.271	47.74±1.89	49.22±3.03	0.677	53.71±4.53	50.29±6.92	0.686
Lymphocytes (%)	20–40	43.15±1.39	44.05±1.54	39.75±3.2	0.211	43.91±1.86	44.40±2.80	0.887	37.88±3.65	44.29±6.6	0.375
Monocytes (%)	2–8	5.2±0.23	5.24±0.27	5.06±0.4	0.746	5.48±0.32	4.63±0.49	0.155	5.49±0.47	4±0.7	0.093
Eosinophils (%)	2–6	1.39±0.17	1.35±0.15	1.55±0.58	0.748	1.35±0.19	1.37±0.25	0.962	1.79±0.8	0.97±0.53	0.537
Basophils (%)	0–1	0.5±0.03	0.5±0.03	0.51±0.08	0.896	0.54±0.04	0.38±0.05	0.010**	0.53±0.11	0.46±0.1	0.711

PLT = Platelet Count; Hb = Hemoglobin; RBC = Red Blood Cell count; HCT = Hematocrit; MCV = Mean Corpuscular Volume; MCH = Mean Corpuscular Hemoglobin; MCHC = Mean Corpuscular Hemoglobin Concentration; WBC = Total White Blood Cells Count.

**: Correlation is significant at the 0.01 level (2-tailed)

*: Correlation is significant at the 0.05 level (2-tailed).

Among 91 DF patients, 65 (71.4%) were male and 26 (28.6%) were female. Gender-based distribution of complete blood and hematological parameters have been presented in Table 1 for patients with DF. It was concluded that the study participants had an almost similar age of 25.6±0.92 years and 26.5±1.27 years in male and female, respectively. However, the female patients had higher levels of platelets (75.15±8.01 K cells/μL) compared to that of male counterparts (58.48±4.86 K cells/μL), which was not statistically significant. All other parameters except platelet count were within the normal reference values as shown in Table 1. Table 1 shows the differences in the other blood parameters between male and female DF patients. Out of 24 patients with DHF, 15 (62.5%) were male and 9 (27.5%) were female. Inferring from Table 1, both male and female participants belong to almost similar age group. Also, the mean level of platelets between the male and female patients with DHF did not vary significantly. Table 1 shows the differences in the other blood parameters between male and female DHF patients.

### Genetic analyses of the exonic regions of GATA1

**Fig 2:**
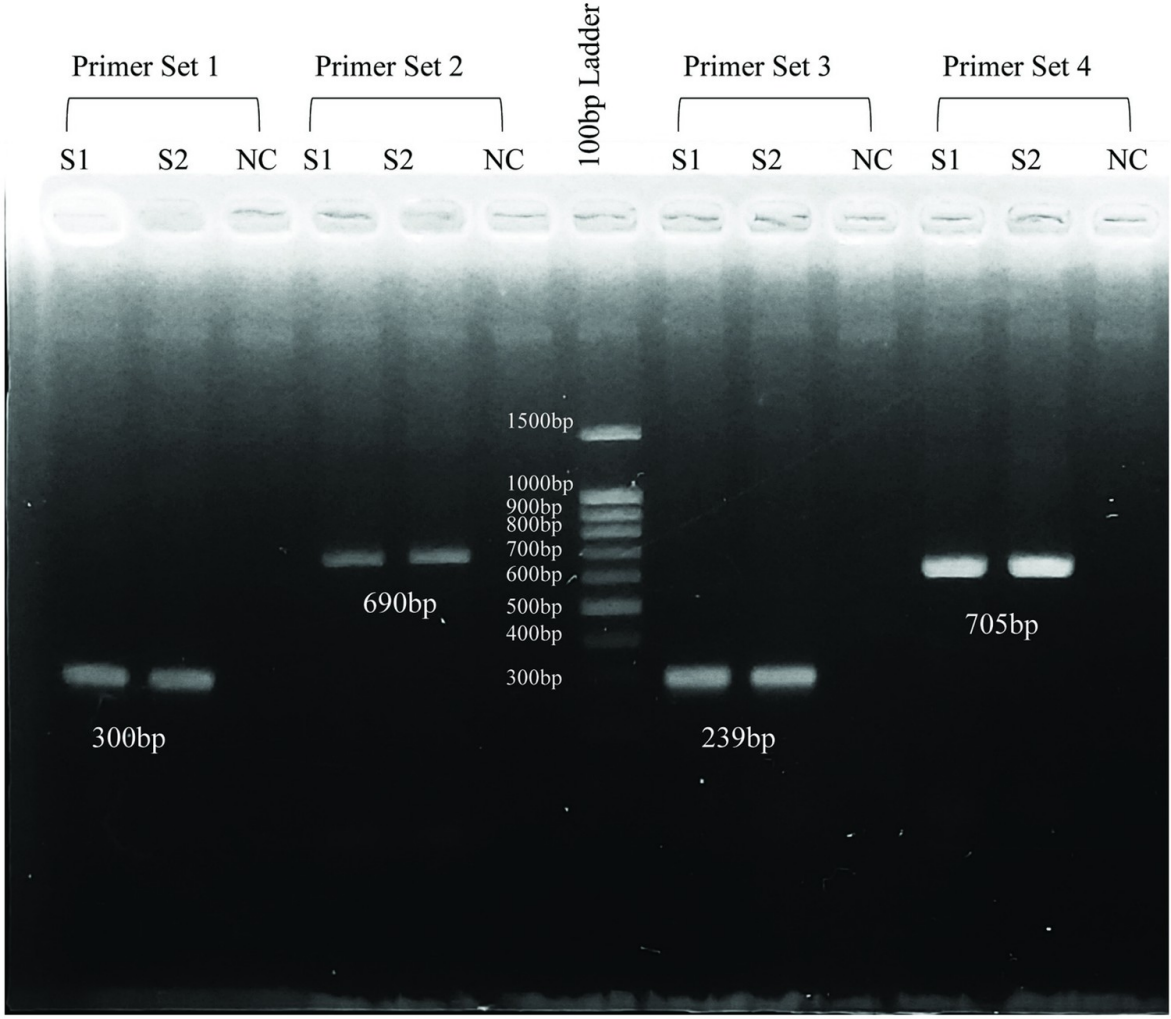
Agarose gel electrophoresis of the amplicons generated from PCR using different primer sets. Primer set 1, 2, 3 and 4 covered the entire coding region of GATA1. For proper estimation of the size of the DNA bands, 100bp ladder DNA was used. *S1 = Sample 1; S2 = Sample 2; NC = Negative Control*.

GATA1 gene is located in the Xp11.23 position of X chromosome. The gene consists of six exons among which exon number 1 acts as promoter region and exon number 2–6 are encoded into mature protein. Four sets of primers (primer sets 1, 2, 3 and 4) targeting and covering exon 2 to 6 of the GATA1 gene were designed. The primers generated amplicons of 300bp, 690bp, 239bp and 705bp for GATA1. The amplicons generated by the primer sets were visualized via agarose gel electrophoresis and have been presented in Fig 2. Upon sequencing the amplicons using Sanger chemistry, the chromatograms were analyzed using the software Geneious Prime (v2020.2.4). The reference sequence NC_000023.11 was retrieved from the NCBI database and the chromatograms were analyzed using Geneious software. In S1A–S1M Fig, the GATA1 variations are found in chromatograms while Table 2 exhibits their respective frequency distribution in total patients, patients with DF and DHF as well as annotation in database.

**Table 2:** Variations in the exonic regions of GATA1 along with their respective frequency distribution in total patients, patients with DF and DHF as well as annotation in database.

Position	Ref Ntd	Mut Ntd	Genotype	Frequency in total participants	Frequency in DF patients	Frequency in DHF patients	Annotation
chX:48791171	C	A	Heterozygous	1 (0.9%)	1 (1.1%)	0 (0%)	Not annotated
chX:48791185	T	A	Heterozygous	2 (1.7%)	2 (2.2%)	0 (0%)	Not annotated
chX:48791244	T	A	Heterozygous	1 (0.9%)	1 (1.1%)	0 (0%)	Not annotated
chX:48791280	T	A	Heterozygous	1 (0.9%)	1 (1.1%)	0 (0%)	Not annotated
chX:48791894	C	T	Heterozygous	1 (0.9%)	1 (1.1%)	0 (0%)	rs937198370
chX:48791918	G	A	Heterozygous	1 (0.9%)	1 (1.1%)	0 (0%)	rs184815507
chX:48791926	G	A	Heterozygous	1 (0.9%)	1 (1.1%)	0 (0%)	rs145355350
chX:48791929	G	T	Heterozygous	2 (1.7%)	2 (2.2%)	0 (0%)	Not annotated
chX:48791947	T	G	Heterozygous	4 (3.5%)	3 (3.3%)	1 (4.2%)	Not annotated
chX:48792009	G	A	Heterozygous	12 (10.43%)	7 (7.7%)	5 (20.8%)	Not annotated
chX:48792118	G	A	Heterozygous	8 (7.0%)	5 (5.5%)	3 (12.5%)	Not annotated
chX:48793213	G	T	Heterozygous	3 (2.6%)	2 (2.2%)	1 (4.2%)	Not annotated
chX:48793292	C	G	Heterozygous	2 (1.7%)	2 (2.2%)	0 (0%)	Not annotated

Ref. Ntd. = Reference Nucleotide; Mut. Ntd. = Mutated Nucleotide.

Chromatogram analyses of the 115 dengue patients, revealed the presence of 13 variants in 22 patients. In 93 patients, no variation was observed at any position when compared to that of the reference sequence and these patients were referred to as “Wild type”. All the 13 variants were heterozygous and found in 22 females which is quite logical as the GATA1 gene is located on the X chromosome. Among these variants, rs937198370 (C>T), rs184815507 (G>A), rs145355350 (G>A) have already been reported in the database while compared with the reference sequence NC_000023.11. Each variant was identified in these dengue patients with a similar frequency of 0.9%. Variants C>A in chX:48791171, T>A in chX:48791185, T>A in chX:48791244, T>A in chX:48791280, G>T in chX:48791929, T>G in chX:48791947, G>A in chX:48792009, C>A in chX:48792118, G>T in chX:48793213 and C>G in chX:48793292 were not annotated in the database and their frequency in the dengue patients were 1 (0.9%), 2 (1.7%), 1 (0.9%),1 (0.9%), 2 (1.7%), 4 (3.5%), 12 (10.43%), 8 (7.0%), 3 (2.6%), and 2 (1.7%), respectively. Among the variants, nine were found in only DF patients while four variants were found in both DF and DHF patients. The variant, G>A (at chX: 48792009) was found in 7.7% of patients with DF and 20.8% of patients with DHF. Another variant, C>A (at chX: 48792118) was present in 5.5% of patients with DF and 12.5% of patients with DHF. Frequencies of these two variants were higher in patients with DHF than DF in this study. Out of 13 variants identified, 6 (46.15%) variants caused synonymous mutations while 7 (53.85%) variants resulted in the change of amino acid residues. Thus, 13 variants had been observed among the study participants and the types of variants, their respective location, annotation change of codon, amino acid substitution, exon number, and mutation type have been presented in Table 3.

**Table 3:** Variations of GATA1 gene and their subsequent amino acid changes in protein sequence.

Type of Variants	Position in X chromosome	Annotation	Codon	Amino acid substitution	Exon number	Types of mutation
C>A	chX:48791171	Not annotated	CCTCaT	P21H	2	Nonsynonymous
T>A	chX:48791185	Not annotated	TCCaCC	S26T	2	Nonsynonymous
T>A	chX:48791244	Not annotated	GCTGCa	A45A	2	Synonymous
T>A	chX:48791280	Not annotated	GCTGCa	A57A	2	Synonymous
C>T	chX:48791894	rs937198370	TCATtA	S91L	3	Nonsynonymous
G>A	chX:48791918	rs184815507	GGCaGC	G99S	3	Nonsynonymous
G>A	chX:48791926	rs145355350	ACGACa	T101T	3	Synonymous
G>T	chX:48791929	Not annotated	GGGGGt	G102G	3	Synonymous
T>G	chX:48791947	Not annotated	ACTACg	T108T	3	Synonymous
G>A	chX:48792009	Not annotated	AGCAaC	S129N	3	Nonsynonymous
C>A	chX:48792118	Not annotated	GGCGGa	G165G	3	Synonymous
G>T	chX:48793213	Not annotated	CAGCAt	Q262H	5	Nonsynonymous
C>G	chX:48793292	Not annotated	CACgAC	H289D	5	Nonsynonymous

Small letters indicate the specific changes in bases that lead to changes in codons.

### Platelet counts in dengue patients with wild type and variants of *GATA1* gene

**Fig 3:**
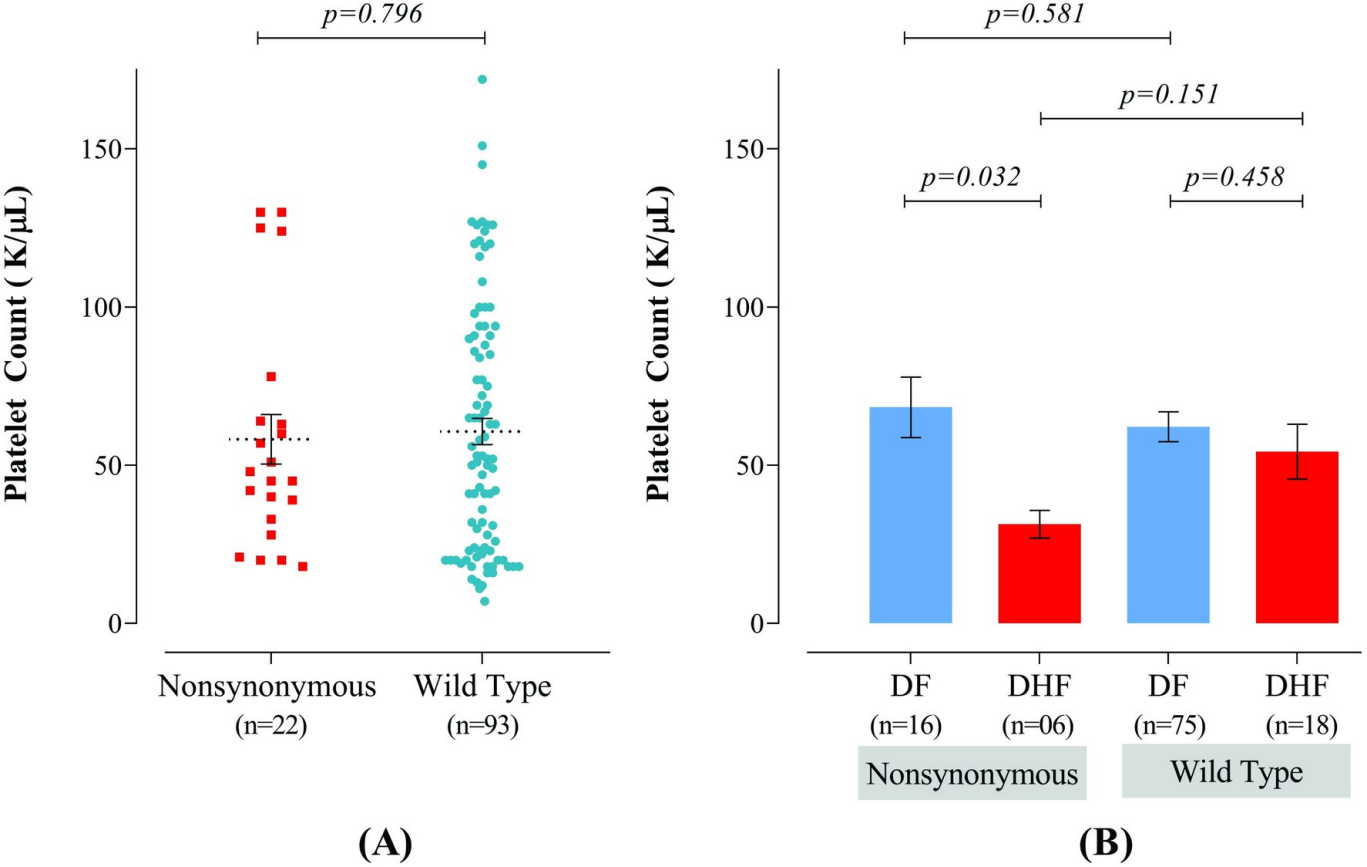
Comparative analyses of the platelet counts. (A) Between different groups of dengue patients with respect to variants recognized within the exonic regions of GATA1 gene with respect to nonsynonymous mutations and wild genotype (those harboring exactly the same sequence as the reference nucleotides). (B) Platelet counts of patients with nonsynonymous and wild type variants in GATA1 gene were stratified according to DF and DHF. Data has been presented as mean*±*SEM and p<0.05 was considered as the level of significance. Statistical analyses revealed that neither of the groups had significant differences.

Fig 3A represents the levels of total platelets between patients with variants and wild type exonic regions of the GATA1 gene. The mean numbers of platelets with nonsynonymous and wild type variants (those harboring the same sequence as the reference nucleotides) were 58.23±7.9 K cells/μL and 60.65±4.14 K cells/μL, respectively. Statistical analyses revealed that the values did not vary significantly between the groups. Further analyses were performed after stratifying the participants into the groups of patients with DF and DHF which have been shown in Fig 3B. The average values of platelets in patients with dengue fever having nonsynonymous and wild type variants were 68.31±9.56 K cells/μL and 62.16±4.7 K cells/μL, respectively. On the other hand, the average value of non-synonymous and wild type variants in patients with dengue hemorrhagic fever was 31.33±4.36 K cells/μL and 54.33±8.68 K cells/μL. Statistical analysis revealed that nonsynonymous variant patients with dengue hemorrhagic fever showed significantly lower platelet count compared to nonsynonymous variant patients with dengue fever (p = 0.032, Fig 3B). Thus, despite there were no significant different in platelet count between patients with non-synonymous mutation genotype and wild genotype, DHF patients with non-synonymous mutation genotype had significantly lower platelet count compared to DF patients with non-synonymous mutation genotype.

### Analyses of platelet counts in dengue patients with respect to highly frequent variants of GATA1 gene

Among the 13 variants observed in the GATA1 gene, G>A variant in chX: 48792009 position and C>A variant in chX: 48792118 position showed a higher frequency of 10.43% and 7.0% in study participants. Moreover, 6 patients were identified who had both the variants.

The platelet count for patients with G>A (at chX:48792009), C>A (at chX:48792118), patients harboring both variants and wild-type variants were 35.08± 4.15 K cells/μL, 31.38± 5.26 K cells/μL, 24.33± 3.25 K cells/μL and 60.65± 4.14 K cells/μL, respectively. The mean difference of platelets in patients harboring G>A variant located at chX: 48792009 and C>A variant located at chX: 48792118 varied significantly from that of the individuals with wild-type nucleotides. These two variants were still significantly associated with reduced platelet counts even after adjusting age and dengue fever type as shown in Table 4. No association of platelet counts with the groups stratified according to mutation was found with or without adjustments (S4 Table). The group that contained individuals harboring zero mutations was used as reference during the analysis by considering it as wild-type. The differences in the mean platelet counts of individuals harboring one, two, three or four mutations and mean platelet counts with respect to individuals harboring no mutations were calculated. Thus, patients harboring the one or both of the two highly frequent G>A (at chX:48792009), C>A (at chX:48792118) mutations, showed statistically lower platelet count compared to wild genotype patients.

**Table 4:** Association of DNA variants with platelets count before and after adjusting the confounding factors in female dengue patients.

Position	Reference Nucleotide	Mutated Nucleotide	Genotypes	*Difference (95% CI)	p-value	*Difference (95% CI)^a+d^	p-value^a+d^
chX:48791171	C	A	Heterozygous	-3.21 (-80.73 to 74.30)	0.94	-6.80 (-84.08 to 70.49)	0.86
chX:48791185	T	A		33.91 (-20.79 to 88.61)	0.23	30.72 (-23.94 to 85.38)	0.27
chX:48791244	T	A		-3.21 (-80.73 to 74.30)	0.94	-6.80 (-84.08 to 70.49)	0.86
chX:48791280	T	A		-3.21 (-80.73 to 74.30)	0.94	-6.80 (-84.08 to 70.49)	0.86
chX:48791894	C	T		-12.29 (-89.77 to 65.19)	0.76	-16.45 (-93.74 to 60.85)	0.68
chX:48791918	G	A		70.43 (-5.99 to 146.85)	0.07	69.46 (-6.94 to 145.85)	0.078
chX:48791926	G	A		70.43 (-5.99 to 146.85)	0.07	69.46 (-6.94 to 145.85)	0.078
chX:48791929	G	T		1.34 (-53.71 to 56.39)	0.96	6.26 (-48.82 to 61.35)	0.82
chX:48791947	T	G		-22.98 (-62.03 to 16.07)	0.25	-23.81 (-62.90 to 15.28)	0.24
chX:48792009	G	A		-28.02 (-50.99 to -5.06)	0.02	-25.53 (-48.86 to -2.19)	0.03
chX:48792118	G	A		-30.96 (-58.67 to -3.25)	0.03	-28.60 (-56.44 to -0.75)	0.04
chX:48793213	G	T		24.80 (-20.12 to 69.72)	0.28	27.76 (-17.07 to 72.59)	0.23
chX:48793292	C	G		32.89 (-21.83 to 87.61)	0.24	31.50 (-23.34 to 86.34)	0.26

*Difference = mean platelet counts with respect to heterozygous mutant–mean platelets count with respect to wild type

a+d = after adjusting age and dengue fever type (DF and DHF).

### Effect of nonsynonymous mutations identified within the exonic regions on the GATA1 protein and their probable association with disease

A total of seven mutations were identified that caused change in amino acid. The impact of the nonsynonymous mutations on the structure of GATA1 was investigated using web-based tools that include SIFT, PolyPhen-2, I-Mutant 2.0, PhD SNP, SNPs&GO and PANTHER-PSEP. SIFT server predicted the variation P21H and H289D to be damaging but the rest 5 mutations were termed as “tolerated”. The variants S26T and H289D were considered to be damaging by the PolyPhen-2 server (Table 5). While the other mutations P21H, S91L, G99S, S129N and Q262H were considered to be benign.

**Table 5:** Prediction of association between GATA1 nonsynonymous mutations and disease.

Mutations	SIFT		PolyPhen-2			
	Score	Prediction	Score	Sensitivity	Specificity	Comments
**P21H**	0.01	Damaging	0.346	0.90	0.89	Benign
**S26T**	1.00	Tolerated	0.948	0.79	0.95	Possibly Damaging
**S91L**	0.70	Tolerated	0.435	0.89	0.90	Benign
**G99S**	0.92	Tolerated	0.000	1.00	0.00	Benign
**S129N**	0.16	Tolerated	0.001	0.99	0.15	Benign
**Q262H**	0.28	Tolerated	0.302	0.91	0.89	Benign
**H289D**	0.00	Damaging	1.000	0.00	1.00	Probably Damaging

A similar approach was used for PhD SNP, SNPs&GO and PANTHER-PSEP tools for predicting effect mutation on GATA1. The data has been presented in S3 Table. It was found that variant H289D was recognized as an outcome of the tools used. Other variants showed varying effects from neutral effects to disease association in different tools. G99S was ‘Damaging’ in PANTHER-PSEP and PhD SNP, while S91L was found to be disease-causing in PhD SNP. Other mutations were found to be neutral. The stability of the mutated proteins was analyzed using I-Mutant 2.0 where S91L and Q262H mutations predicted to increase the stability of the protein and the rest of the variants showed a decrease in stability. Thus, through different in silico analysis, it had been observed that the non-synonymous S26T, S91L, G99S, H289D had the potential of disease progression in most web-based platforms.

### Analyses of the impact of the mutation on the three-dimensional structure of GATA1 protein

Amino acid sequence of human GATA1 was retrieved from UniProt available at www.uniprot.org (ID: P15976). The three-dimensional structure of GATA1 was predicted using *ab initio* modelling approach using RaptorX structure prediction web-based platform.

**Fig 4:**
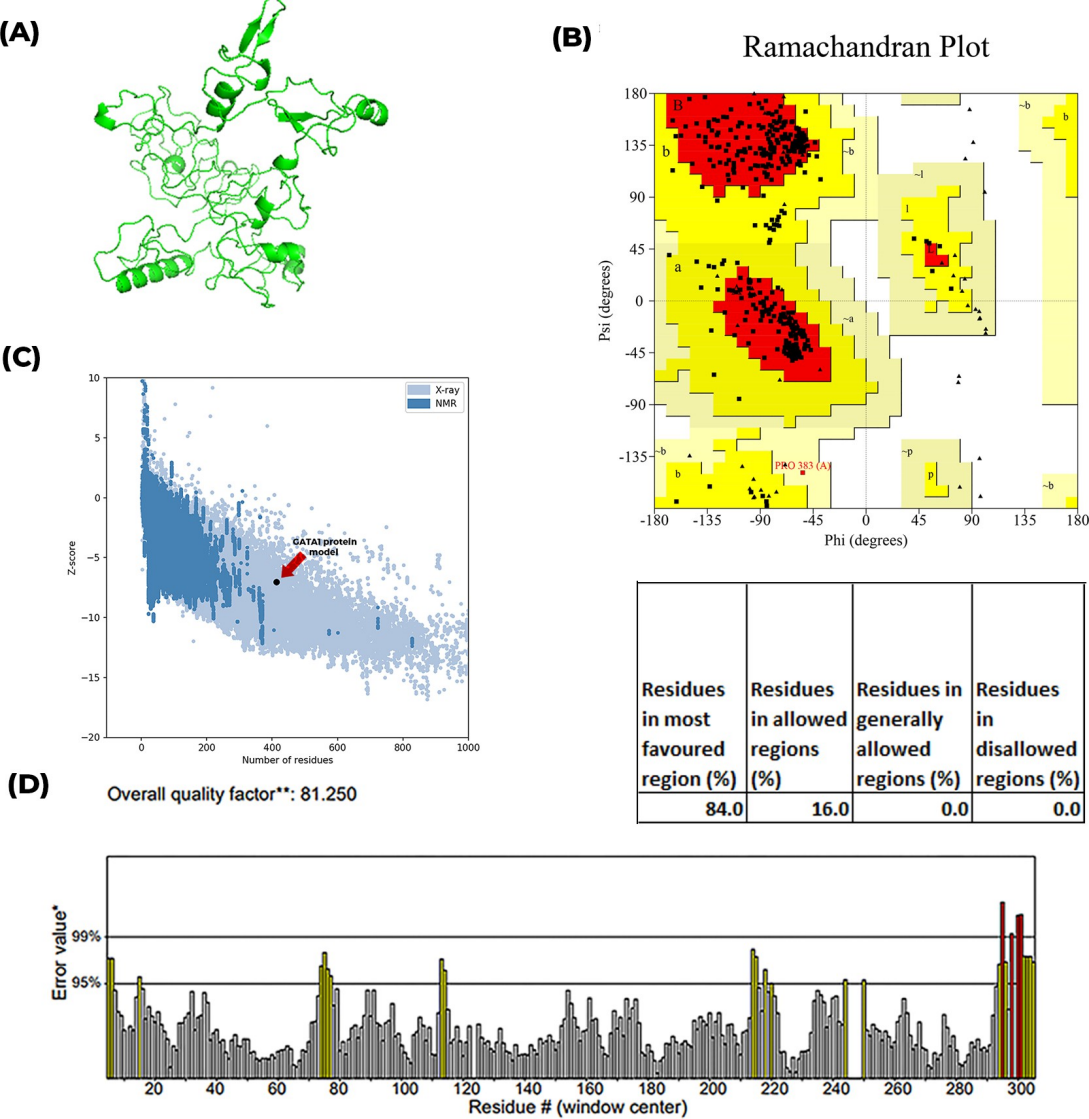
Structure analyses of *ab initio* GATA1 modelled protein. (A) Predicted 3D structure of the protein (B) Ramachandran plot of the structure (C) Z-score plot from PROSA (D) Residual error value plot from ERRAT2.

In PROSA, the GATA1 protein was placed in the “X-ray” region with a Z score of -6.86. This interpreted that the modelled structure had a similar structure to the protein whose three- dimensional structure has been discovered by X-ray crystallography method. From using ERRAT2, 81.25% score was achieved for the GATA1 protein model. The score was considered low, as good high-resolution structures generally produce values of more than 95%. Overall, the modelled structure of GATA1 has been considered for use as the original structure has not yet been resolved by NMR or X-ray crystallography method. Fig 4 represents the prediction of the three-dimensional structure of GATA1 protein followed by its validation. The quality of the predicted structure was checked using PROCHECK, Ramachandran Plot, PROSA and ERRAT2 web-based software. From PROCHECK, structure analyses in the Ramachandran plot revealed that a total of 84% amino acid residues of the predicted structure of protein were within the most favored region while 16% of them laid within the additional allowed region. A good quality model would be expected to have over 90% in the most favored region.

The modelled structure was then used as a template in Missense3D to measure the Relative Solvent Accessibility (RSA), and cavity volume alteration of the mutated amino acids. For P21H mutation, the cavity volume was contracted by 98.28 Å^3^ (angstrom cube, a unit to measure volume). Since it is more than 70 Å^3^, this mutation can alter the protein structure. G99S mutation resulted in structural alteration also due to replacement of structural glycine amino acid. However, no significant RSA change in the mutational amino acids occurred to be considered as a protein structure modifier from MisSense3D. Thus, according to Missense3D, the nonsynonymous mutations P21H and G99S had the potential to generate structural alteration of GATA1 protein and affect the function of the protein.

### Comparative three-dimensional structural analyses between wild type and mutant protein of GATA1

**Fig 5:**
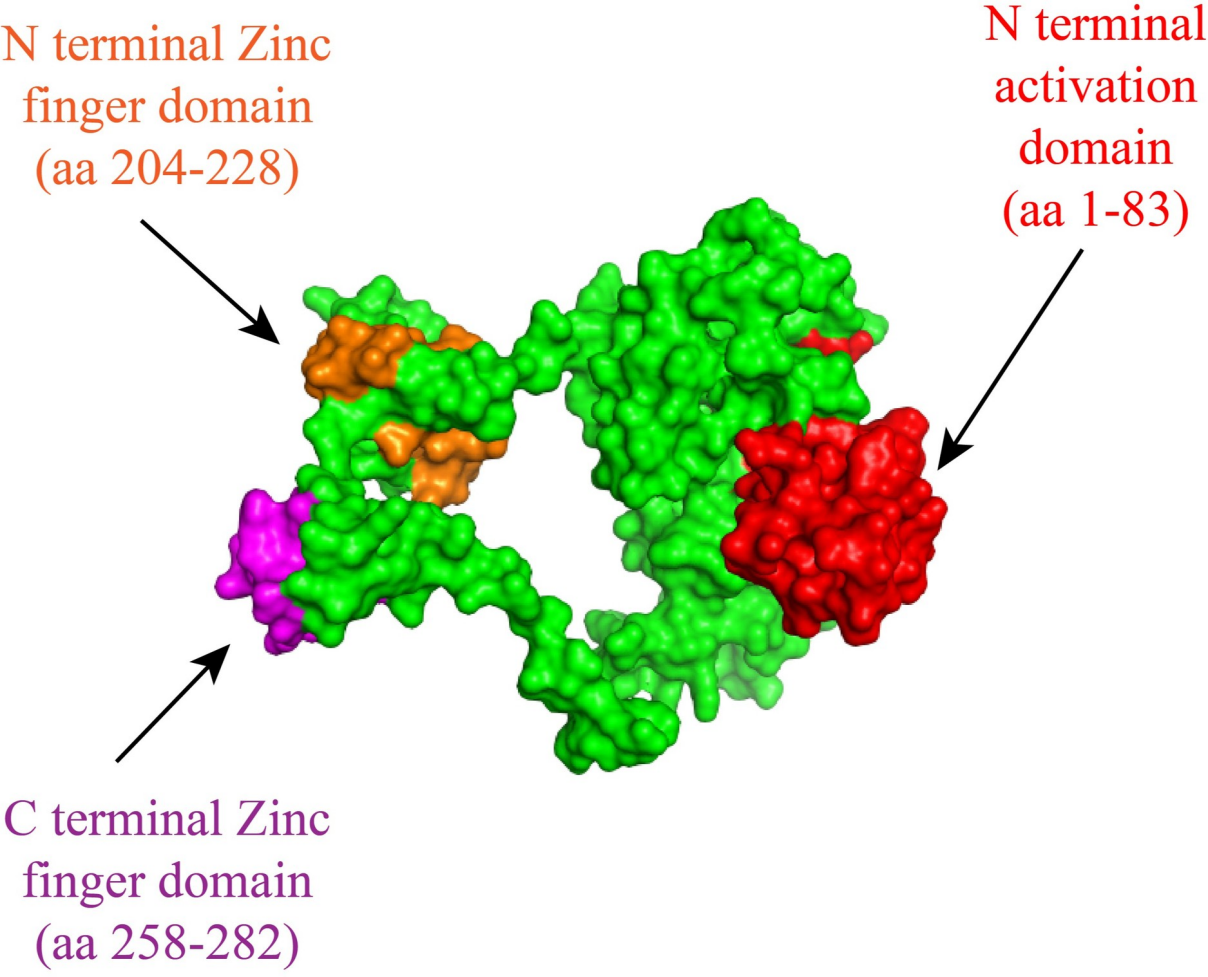
Surface structure of the modelled GATA1 protein with structural domains. N-terminal activation domain, AD (red color), N-terminal Zinc finger domain, N-ZF (orange color) and C terminal Zinc finger domain, C-ZF (purple color).

Computational homology modelling tool, Modeller, was applied to generate mutated GATA1 protein models with variations found in this study and the modelled structures were refined using GalaxyWeb, a protein structure prediction and refinement web server. The mutated structures were then aligned with the modelled structure using Pymol Software and Root-Mean-Square Deviation (RMSD) score for the mutation was achieved. The surface structure along with the functional domains are presented in Fig 5. Total 7 non-synonymous mutations were observed in the *GATA1* gene. Among them, P21H and S26T mutations were found in the AD domain of the protein while Q262H was within the C-ZF domain. Other mutations including S91L, G99S, S129N and H289D were found in regions outside the functional domains of GATA1 protein. The RMSD value along with change in interaction with neighboring amino acids for the variants is presented in Table 6.

**Table 6:** Structure analyses of mutated amino acids in GATA1 protein using Pymol and Missense3D.

Variations	RMSD score	RSA value			Cavity volume alteration	Structure Alteration
		Wild AA	Mutant AA	Change of RSA		
P21H	0.006	10.20%	5.40%	4.8%	Contracted by 98.28 Å^3^	^#^Alteration
S26T	0.005	91.50%	96.4%.	4.9%	Expand by 1.512 Å^3^	No alteration
S91L	0.005	63.80%	68.20%	4.4%	Contracted by 30.672 Å^3^	No alteration
G99S	0.005	2.30%	3.8%.	1.5	Contracted by 13.176 Å^3^	^##^Alteration
S129N	0.005	32.30%	37.50%	5.2%	-	No alteration
Q262H	0.005	81.80%	94.00%	12.2%	-	No alteration
H289D	0.005	76.00%	69.30%	6.7%	-	No alteration

^#^Cavity alteration more than 70 Å^3^ (angstrom cube, a unit of volume) is conceded structure alteration;

^##^Amino acid change of Glycine is conceded structure alteration.

^###^Difference of RSA value more than 20% is conceded structure alteration.

**Fig 6:**
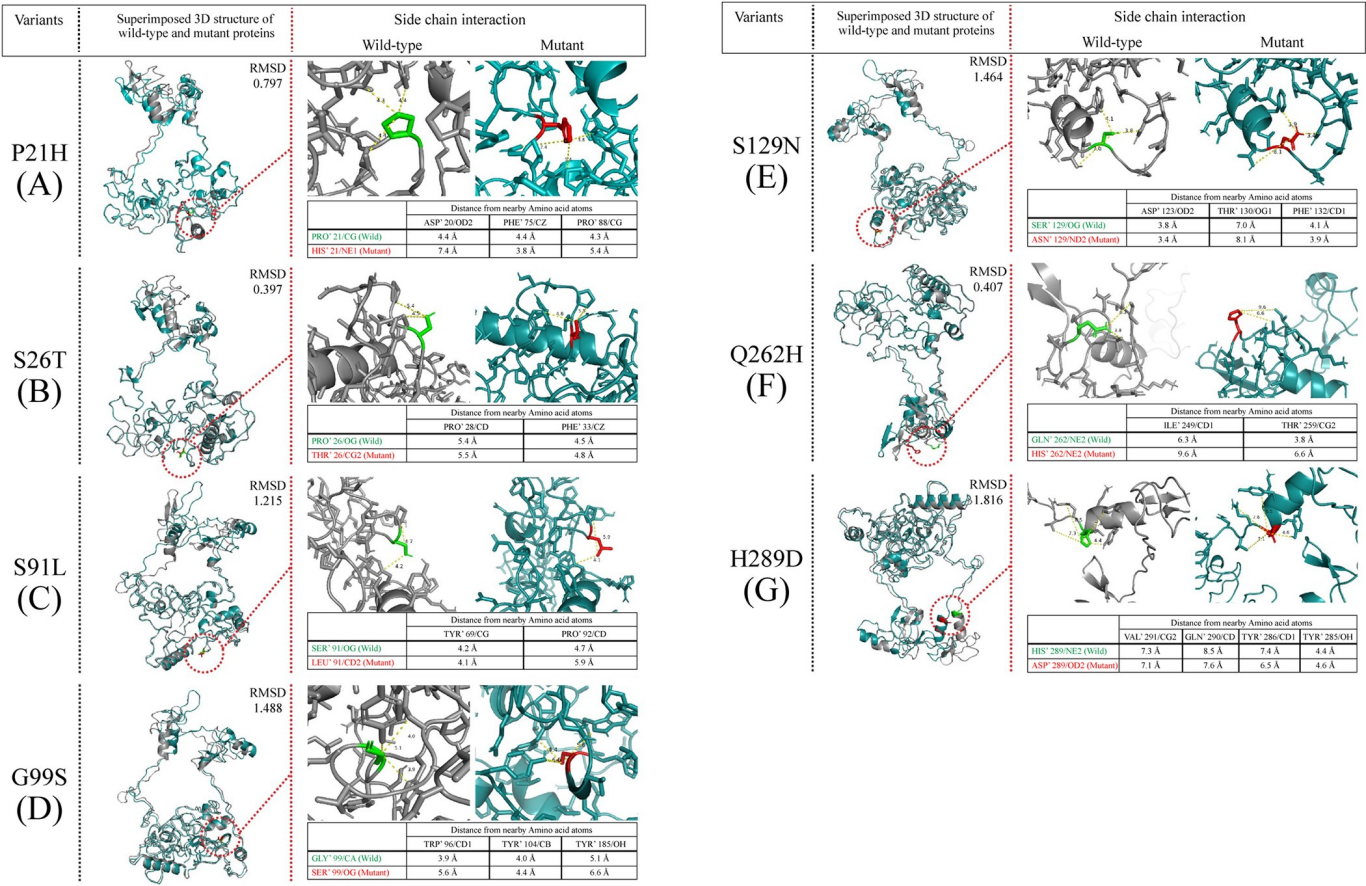
Evaluation of the effect of nonsynonymous mutations on the structure and interactions between side chain and neighboring residues. Among non-synonymous mutations, P21H (A) and S26T (B) mutations were found in the AD domain of the protein while Q262H (F) was within the C-ZF domain. Other mutations including S91L (C), G99S (D), S129N (E) and H289D (G) were found in regions outside the functional domains of GATA1 protein.

The substitution of proline by histidine at position 21 within AD domain has resulted in relaxing the structure of the domain as the distance with nearby interacting amino acids Asp20 and Pro88 increased for mutant amino acid while the RMSD value after superimposition with wild-type GATA1 structure was 0.797. The distance of of Pro21 (wild type) with nearby Asp20 and Pro88 residues were 4.4 Å and 4.3 Å while for His21 (mutant) the values were 7.4 Å and 5.4 Å respectively (Fig 6A). For S26T mutation, much change in structure has not been observed and the distance with nearby residue (Pro28 and Phe33) remained almost the same while wild type as well as mutant amino acid was compared and the RMSD score also remained low when superimposed (Fig 6B). S91L mutant showed higher structure relaxation and the distances with neighboring amino acids (Tyr69 and Pro92) was increased and has been shown in Fig 6C and the RMSD score was 1.215.

The mutation G99S was placed inside the protein structure and the distance of glycine in 99th position with neighboring Trp96, Tyr104 and Tyr185 residues were 3.9 Å, 4.0 Å and 5.1 Å as seen in Fig 6D. The distances increased to 5.6 Å, 4.4 Å and 6.6 Å due to substitution of serine with glycine, respectively. The surface structure for G99S mutation resulted in causing the alteration of the structure with a RMSD score of 1.488 when the structures were superimposed. The distance for mutant asparagine at position 129 with nearby Asp123, Thr130 and Phe132 residues were 3.4 Å, 8.1 Å and 3.9 Å, respectively. Replacement of asparagine with serine decreased the distances to 3.8 Å, 7.0 Å and 4.1 Å for wild GATA1 protein structure, accordingly (Fig 6E) and the RMSD value was 1.464 for the mutant. The Q262H mutant was found within the C terminal Zinc finger domain (C-ZF). Substation of glutamine with histidine resulted in expansion of the surface volume. The distance of glutamine with neighboring residues Ile249 and Thr259 were 6.3 Å and 3.8 Å respectively, which increased to 9.6 Å and 6.6 Å for substituted histidine residue, as shown in Fig 6F. However, the RMSD score was low when the mutant Q262H was superimposed with wild GATA1 modelled structure. The position of H289D mutation was near the end of the C-ZF domain but the mutation did not vary the structure much as seen from the Fig 6G. The distance between wild and mutant amino acids with nearby Val291, Gln290, Tyr286 and Tyr285 didn’t had much difference.

## Discussion

In 2019, Bangladesh encountered its largest dengue virus (DENV) epidemic affecting more than a hundred thousand people. Although most patients were asymptomatic, a large sum of patients demonstrated dengue fever (DF) along with dengue hemorrhagic fever (DHF)/dengue shock syndrome (DSS) with different degrees of thrombocytopenia. However, the mechanisms and host genetic factors that lead to thrombocytopenia during dengue infection and eventually DHF have not been determined, and a great challenge in the early identification of patients who are more likely to progress to a worse health condition. This study was aimed to investigate potential variations within entire exonic regions of GATA1 gene, one of the master transcription factor for platelet production, in dengue patients with thrombocytopenia and thus, establishes a relationship between gene variants and disease severity.

Although all study participants had thrombocytopenia, DHF patients showed a lower mean of platelets than that of DF, which was statistically insignificant. However, thrombocytopenia is a constant feature and one of the diagnostic criteria of DHF. A study on the Brazilian population also supported that patients with DHF had significantly lower platelet counts than patients with DF [40]. Further, though patients had bleeding manifestations, they did not develop any symptoms of plasma leakage which could ultimately lead to dengue shock syndrome as reflected by the values of hematocrit and hemoglobin which were found to be within the reference ranges. Parameters of patients with DF and DHF were also compared independently on the basis of gender as shown in Table 1. Hematocrit, hemoglobin and levels of RBC showed statistically significant differences between male and female individuals in the groups of patients with DF and DHF but the mean values of these parameters were found to be within the reference ranges in both genders.

For analyzing genetic variations within the GATA1, all exonic regions covering exon 2 to 6 of the GATA1 gene were amplified using template genomic DNA from all the 115 patients. Exon 1 was excluded as it contains promoter regions for the gene and thus, it remains untranslated. The chromatograms obtained through Sanger sequencing were subjected to further analyses to identify the genetic variations. From the analysis, 13 variants were identified in 22 female patients and all the mutants were heterozygous. No heterozygosity were observed in male patients and this is because GATA1 is located in chromosome X. In fact no polymorphisms were observed in any of the 13 positions where variants were observed in females. Since no polymorphism was observed in males and none of the mutants were homozygous in females, there is a possibility that these mutations are deleterious in nature. According to Ensembl (https://asia.ensembl.org/) database, a total of 6334 single nucleotide polymorphisms (SNPs) have been reported to be identified within the GATA1 gene, among which 537 are missense variants. Till to date, a total of 66 SNPs within GATA1 have been reported to be associated with different diseases including thrombocytopenia and Diamond-Blackfan anemia [41]. Among them, V205M, G208R, D218Y, G208S, D218G, D218N, R216Q, R216W, R224L, D237H, Q237H, S329R, G356V have been reported to be associated with thrombocytopenia [22,42–47]

However, variations concerning other amino acids have not been elucidated yet and the high frequent variants observed from this study may have the potential to exacerbate thrombocytopenia during dengue infection. Out of 13 variants, 7 were non-synonymous and 6 were synonymous. The variations found in the exons 2, 3 and 5 of the GATA1 gene. While 3 variants (one nonsynonymous and two synonymous) were found to be reported in the NCBI database, 10 were recognized as new variants. Among the annotated variants, no disease association was revealed so far. Variants G>A at chX: 48792009 and C>A at chX: 48792118 were found as high frequent variants and were respectively present in 10.43% and 7.0% of the total study population. The subsequent changes of amino acids in GATA1 protein due to the mutations recognized have been displayed in Table 3. The variants with the highest frequencies, G>A at chX: 48792009 and C>A at chX: 48792118 showed nonsynonymous (S129N) and synonymous effects, respectively. The nonsynonymous variants were further analyzed to investigate their probable impacts on the functions of proteins using different web-based approaches and it was revealed that only substitution of histidine by glutamic acid at position 289 (H289D) showed to be deleterious with respect to all the tools used in the study as shown in Tables S3 and 5. This may be caused by the complete shift of charge from positive to negative that led to changed interaction pattern between the neighboring amino acids followed by the biological function of protein.

Later, the three-dimensional structure of GATA1 was modelled using RaptorX and then, quality of the modelled structure was evaluated through different *in silico* platforms that has been portrayed in Fig 4A. The modelled protein had 84% residues in the most favorable region according to the Ramachandran plot (Fig 4B) and Z-Score was found to be -6.86 (Fig 4C). A well-structured model should have more than 95% residues in the most favorable region. But proteins whose full structure has not yet been resolved from X-ray crystallography or NMR, computational structures that have more than 80% residues in the most favorable region of the Ramachandran plot can be considered as a good structure [48]. As the predicted structure of protein had more than 80% residues in the desired region and the Z score lied in the region with established structures, the modelled structure was subjected to further analyses. Further, the ERRAT2 (a measure of the quality of non-bonded interactions) quality score 81.25 (Fig 4D) reassured the overall quality of the modelled structure as well as reliability of the prediction. The impact of the mutations on the three-dimensional structure of the protein was first analyzed in MisSense3D and later, the surface structure, as well as side chain interactions, were analyzed using Pymol.

The nonsynonymous variant with high frequency (G>A at chX: 48792009) was present in 7.7% of patients with dengue fever and in 20.8% patients with dengue hemorrhagic fever. It conferred a nonsynonymous effect on GATA1 by changing the codon (AGC>AaC) and replacing serine with asparagine at the 129^th^ amino acid position. Though, variant S129N was predicted to be neutral in different *in silico* approaches but patients harboring S129N mutation had a significantly (p = 0.02) lower platelet count (35.08±4.15 Kcells/μL) compared to the platelets of patients (60.65±4.14 Kcells/μL) carrying wild-type nucleotide as that present in the reference sequence of GATA1 (NC_000023.11) and same result was observed even after adjusting age and dengue fever type (Table 4). However, I-Mutant 2.0 predicted that the mutation tends to increase the stability of the protein. The three-dimensional structure analyses from Fig 6E, revealed that the substitution of serine to asparagine leads to a change in the structure of the protein GATA1 with a RMSD score of 1.464. The distance of wild type amino acid (Ser129) from nearby Asp123, Thr130 and Ser131 residues were 3.8 Å, 7.0 Å and 4.1Å. But the distances reduced to 3.4 Å and 3.9 Å for Asp123 and Phe 132 while increased about 1.1 Å for Thr130 when serine was substituted by asparagine. Though serine and asparagine are uncharged polar amino acids, the side chain of asparagine (-CH2- CO-NH2) is relatively large compared to that of serine (-CH2-OH). Thus, asparagine was shown to be more exposed towards outer surface compared to serine and decreased the cavity volume. As from Fig 6E, serine at position 129 (Ser129) lies close to the N-terminal activation domain of GATA1 protein and mutation to asparagine can hamper the function of the domain by affecting the interaction pattern with the nearby residues. The function of the N-terminal activation domain is to form homodimer as well as heterodimer with GATA2 [49]. We hypothesized that increased stability (S3 Table) of the protein due to substitution of serine by asparagine may cause increased rigidity followed by reducing structural flexibility which may ultimately disturb the dimerization process of the GATA1 protein. This, in turn, may affect the function followed by impaired production of mature platelets which may be reflected by the lower platelet counts in individuals with such variation.

A synonymous (GGC-GGa) variant with high frequency obtained from this study was C>A at chX: 48792118. The variation was present in 5.5% of dengue fever and 12.5% of dengue hemorrhagic fever participants (Table 2). Patients with this variant had significantly (p = 0.03 and 0.04, respectively before and after adjustments, Table 4) lower platelet count (31.38±5.26 K cells/μL) compared to that of platelet count (60.65±4.14 Kcells/μL) measured in patients who harbored reference nucleotide (NC_000023.11) at the same position. It is important to note that even synonymous variations can lead to altered expression of protein due to codon bias [50]. From, GenScript Codon Usage Frequency Table Tool (www.genscript.com), the frequency of the wild-type codon GGC is 34% while the mutant codon GGa is 25% for *Homo sapiens*. The more frequent the codon usage is, the higher the availability of the respective tRNA is found during protein production [50]. Thus, change to a less frequent codon can decrease the production of GATA1 protein due to the lower availability of tRNA which respond to the changed codon. As a result, synonymous variation to less frequent codon could lead to altered expression of GATA1 protein which eventually may affect thrombopoiesis followed by thrombocytopenia. Furthermore, the mean value of platelet measured in 6 patients harboring both the high frequent variants (G>A at chX:48792009 and C>A at chX:48792118) was 24.33±3.25 K cells/μL which was lower than the individuals having either of the single variants. This suggests that patients with these two variants are more prone to thrombocytopenia. Impact of these variants on the activation domain of GATA1 and probable effect on the expression of GATA1 may support the underlying cause of clinical outcome of the study participants reflected by the values of platelets.

## Conclusion

In this study, new variants have been identified in the exonic regions of GATA1 gene. Of them, G>A at chX: 48792009 and C>A at chX: 48792118 were highly frequent and patients harboring any one or both of the mutations showed severe thrombocytopenia. From, *in silico* analyses it was also observed that the nonsynonymous mutation exerted by G>A at chX: 48792009 had further impact on the structure and function of GATA1 protein. Thus, due to the importance of the GATA1 gene in thrombopoiesis and in dengue severity, this study needs to be further validated in a large number of populations residing in different geographical regions.

## Supporting information

S1 Fig Variations found in the coding region of GATA1 gene. Representing chromatograms obtained through Sanger sequencing of the entire exonic regions of GATA1 gene demonstrating genotypic variations. It has been recognized that all the frequencies represented the heterozygous genotype.
(TIF)

S1 Table List of primers designed using Primer3 web based tool to amplify the exonic regions of GATA1 gene. (DOCX)

S2 Table Optimum conditions applied to perform polymerase chain reaction to amplify targeted regions of GATA1. (DOCX)

S3 Table Prediction of association between GATA1 nonsynonymous mutations and along with their effects and on the stability of the protein. (DOCX)

S4 Table Association of mean platelet counts with the number of variants harbored by the patients before and after adjusting the confounding factors in female dengue patients. (DOCX)

## References

[1] Bhatt S, Gething PW, Brady OJ, Messina JP, Farlow AW, Moyes CL, et al. The global distribution and burden of dengue. Nature. 2013;496: 504–507. doi: 10.1038/nature12060 23563266 PMC3651993

[2] Tjaden NB, Thomas SM, Fischer D, Beierkuhnlein C. Extrinsic {Incubation} {Period} of {Dengue}: {Knowledge}, {Backlog}, and {Applications} of {Temperature} {Dependence}. PLoS Negl Trop Dis. 2013;7. doi: 10.1371/JOURNAL.PNTD.0002207 23826399 PMC3694834

[3] Srikiatkhachorn A. Plasma leakage in dengue haemorrhagic fever. Thromb Haemost. 2009;102: 1042–1049. doi: 10.1160/TH09-03-0208 19967133 PMC5527705

[4] Tsai C-J -J, Kuo C-H -H, Chen P-C -C, Changcheng C-S -S. Upper gastrointestinal bleeding in dengue fever. Am J Gastroenterol. 1991;86: 33–35. doi: 10.1111/j.1572-0241.1991.tb06824.x 1986551

[5] Kayal L, Jayachandran S, Singh K. Idiopathic thrombocytopenic purpura. Contemp Clin Dent. 2014;5: 410–414. doi: 10.4103/0976-237X.137976 25191085 PMC4147825

[6] Cines DB, Blanchette VS. Immune thrombocytopenic purpura. N Engl J Med. 2002;346: 995–1008. doi: 10.1056/NEJMra010501 11919310

[7] Clinical {Management} and {Delivery} of {Clinical} {Services}, {Dengue}: {Guidelines} for {Diagnosis}, {Treatment}, {Prevention} and {Control}. Available: www.who.int/tdr

[8] Ojha A, Nandi D, Batra H, Singhal R, Annarapu GK, Bhattacharyya S, et al. Platelet activation determines the severity of thrombocytopenia in dengue infection. Sci Rep. 2017;7. doi: 10.1038/SREP41697 28139770 PMC5282509

[9] Azeredo EL De Monteiro RQ, Pinto LMD-O. Thrombocytopenia in {Dengue}: {Interrelationship} between {Virus} and the {Imbalance} between {Coagulation} and {Fibrinolysis} and {Inflammatory} {Mediators}. Mediators Inflamm. 2015;2015. doi: 10.1155/2015/313842 25999666 PMC4427128

[10] Hottz ED, Oliveira MF, Nunes PCG, Nogueira RMR, Valls-de-Souza R, Poian AT Da, et al. Dengue induces platelet activation, mitochondrial dysfunction and cell death through mechanisms that involve {DC}-{SIGN} and caspases. J Thromb Haemost. 2013;11: 951–962. doi: 10.1111/jth.12178 23433144 PMC3971842

[11] Assinger A. Platelets and infection—an emerging role of platelets in viral infection. Front Immunol. 2014;5. doi: 10.3389/FIMMU.2014.00649 25566260 PMC4270245

[12] Srichaikul T, Nimmannitya S, Sripaisarn T, Kamolsilpa M, Pulgate C. Platelet function during the acute phase of dengue hemorrhagic fever. Southeast Asian J Trop Med Public Health. 1989;20: 19–25. Available: https://pubmed.ncbi.nlm.nih.gov/2772702/ 2772702

[13] Xavier-Carvalho C, Cardoso CC, Kehdy F de S, Pacheco AG, Moraes MO. Host genetics and dengue fever. Infect Genet Evol. 2017;56: 99–110. doi: 10.1016/j.meegid.2017.11.009 29133029

[14] Johnson B, Fletcher SJ, Morgan N V. Inherited thrombocytopenia: novel insights into megakaryocyte maturation, proplatelet formation and platelet lifespan. Platelets. 2016;27: 519–525. doi: 10.3109/09537104.2016.1148806 27025194 PMC5000870

[15] Schulze H, Shivdasani RA. Mechanisms of thrombopoiesis. J Thromb Haemost. 2005;3: 1717–1724. doi: 10.1111/j.1538-7836.2005.01426.x 16102038

[16] Geddis AE. Megakaryopoiesis. Semin Hematol. 2010;47: 212–219. doi: 10.1053/j.seminhematol.2010.03.001 20620431 PMC2904992

[17] Ito E, Toki T, Ishihara H, Ohtani H, Gu L, Yokoyama M, et al. Erythroid transcription factor {GATA}-1 is abundantly transcribed in mouse testis. Nature. 1993;362: 466–468. doi: 10.1038/362466a0 8464479

[18] Martin DIK, Orkin SH. Transcriptional activation and {DNA} binding by the erythroid factor {GF}-1/{NF}-{E1}/{Eryf} 1. Genes \& Dev. 1990;4: 1886–1898. doi: 10.1101/gad.4.11.1886 2276623

[19] Yang HY, Evans T. Distinct roles for the two {cGATA}-1 finger domains. Mol Cell Biol. 1992;12: 4562–4570. doi: 10.1128/mcb.12.10.4562-4570.1992 1406646 PMC360383

[20] Lecine P, Villeval JL, Vyas P, Swencki B, Xu Y, Shivdasani RA. Mice {Lacking} {Transcription} {Factor} {NF}-{E2} {Provide} {In} {Vivo} {Validation} of the {Proplatelet} {Model} of {Thrombocytopoiesis} and {Show} a {Platelet} {Production} {Defect} {That} {Is} {Intrinsic} to {Megakaryocytes}. Blood. 1998;92: 1608–1616. doi: 10.1182/BLOOD.V92.5.1608 9716588

[21] Kwiatkowski BA, Bastian LS, Bauer TR, Tsai S, Zielinska-Kwiatkowska AG, Hickstein DD. The ets family member {Tel} binds to the {Fli}-1 oncoprotein and inhibits its transcriptional activity. J Biol Chem. 1998;273: 17525–17530. doi: 10.1074/jbc.273.28.17525 9651344

[22] Millikan PD, Balamohan SM, Raskind WH, Kacena MA. Inherited thrombocytopenia due to {GATA}-1 mutations. Semin Thromb Hemost. 2011;37: 682–689. doi: 10.1055/s-0031-1291378 22102271

[23] Chakraborty S, Alam S, Sayem M, Sanyal M, Das T, Saha P, et al. Investigation of the efficacy and safety of eltrombopag to correct thrombocytopenia in moderate to severe dengue patients—a phase {II} randomized controlled clinical trial. EClinicalMedicine. 2020;29–30. doi: 10.1016/j.eclinm.2020.100624 33294822 PMC7691733

[24] Dengue: guidelines for diagnosis, treatment, prevention and control. {World} {Health} {Organization}. World Heal Organ Spec Program Res Train Trop Dis World Heal Organ. 2009. Available: https://apps.who.int/iris/handle/10665/44188 23762963

[25] Kalayanarooj S. Clinical {Manifestations} and {Management} of {Dengue}/{DHF}/{DSS}. Trop Med Health. 2011;39: 83–87. doi: 10.2149/tmh.2011-S10 22500140 PMC3317599

[26] Srikiatkhachorn A, Rothman AL, Gibbons R V, Sittisombut N, Malasit P, Ennis FA, et al. Dengue–how best to classify it. Clin Infect Dis. 2011;53: 563–567. doi: 10.1093/cid/cir451 21832264 PMC3202316

[27] N H, MI H, Y T, S PK, H AKMM, N AHMN. Genetic variation of the transcription factor GATA3, not STAT4, is associated with the risk of type 2 diabetes in the Bangladeshi population. PLoS One. 2018;13. doi: 10.1371/JOURNAL.PONE.0198507 30044774 PMC6059405

[28] Bappy HMJA, Goswami A, Huda N, Hosen MI, Nabi AHMN. Gender specific association of missense variant rs1805097 of IRS-2 and noncoding variant rs841853 of GLUT-1 genes with susceptibility to type 2 diabetes in Bangladeshi population. Gene Reports. 2020;21. doi: 10.1016/J.GENREP.2020.100866

[29] A G, N H, T Y, MI H, AKMM H, AHMN N. Association study of leukocyte telomere length and genetic polymorphism within hTERT promoter with type 2 diabetes in Bangladeshi population. Mol Biol Rep. 2021;48: 285–295. doi: 10.1007/s11033-020-06045-7 33389530

[30] Koressaar T, Remm M. Enhancements and modifications of primer design program {Primer3}. Bioinformatics. 2007;23: 1289–1291. doi: 10.1093/bioinformatics/btm091 17379693

[31] Tsai SF, Strauss E, Orkin SH. Functional analysis and in vivo footprinting implicate the erythroid transcription factor {GATA}-1 as a positive regulator of its own promoter. Genes \& Dev. 1991;5: 919–931. doi: 10.1101/gad.5.6.919 2044960

[32] Andreson R, Puurand T, Remm M. {SNPmasker}: automatic masking of {SNPs} and repeats across eukaryotic genomes. Nucleic Acids Res. 2006;34: W651—655. doi: 10.1093/nar/gkl125 16845091 PMC1538889

[33] Sim N-L, Kumar P, Hu J, Henikoff S, Schneider G, Ng PC. {SIFT} web server: predicting effects of amino acid substitutions on proteins. Nucleic Acids Res. 2012;40: W452—W457. doi: 10.1093/nar/gks539 22689647 PMC3394338

[34] Adzhubei IA, Schmidt S, Peshkin L, Ramensky VE, Gerasimova A, Bork P, et al. A method and server for predicting damaging missense mutations. Nat Methods. 2010;7: 248–249. doi: 10.1038/nmeth0410-248 20354512 PMC2855889

[35] Calabrese R, Capriotti E, Fariselli P, Martelli PL, Casadio R. Functional annotations improve the predictive score of human disease-related mutations in proteins. Hum Mutat. 2009;30: 1237–1244. doi: 10.1002/humu.21047 19514061

[36] Capriotti E, Calabrese R, Casadio R. Predicting the insurgence of human genetic diseases associated to single point protein mutations with support vector machines and evolutionary information. Bioinformatics. 2006;22: 2729–2734. doi: 10.1093/bioinformatics/btl423 16895930

[37] Tang H, Thomas PD. {PANTHER}-{PSEP}: predicting disease-causing genetic variants using position-specific evolutionary preservation. Bioinformatics. 2016;32: 2230–2232. doi: 10.1093/bioinformatics/btw222 27193693

[38] Bava KA, Gromiha MM, Uedaira H, Kitajima K, Sarai A. {ProTherm}, version 4.0: thermodynamic database for proteins and mutants. Nucleic Acids Res. 2004;32: D120—121. doi: 10.1093/nar/gkh082 14681373 PMC308816

[39] Sali A, Blundell TL. Comparative protein modelling by satisfaction of spatial restraints. J Mol Biol. 1993;234: 779–815. doi: 10.1006/jmbi.1993.1626 8254673

[40] Mourão MPG, Lacerda MVG, Macedo VO, Santos JB. Thrombocytopenia in patients with dengue virus infection in the {Brazilian} {Amazon}. Platelets. 2007;18: 605–612. doi: 10.1080/09537100701426604 18041652

[41] Crispino JD, Horwitz MS. {GATA} factor mutations in hematologic disease. Blood. 2017;129: 2103–2110. doi: 10.1182/blood-2016-09-687889 28179280 PMC5391620

[42] Nichols KE, Crispino JD, Poncz M, White JG, Orkin SH, Maris JM, et al. Familial dyserythropoietic anaemia and thrombocytopenia due to an inherited mutation in {GATA1}. Nat Genet. 2000;24: 266–270. doi: 10.1038/73480 10700180 PMC10576470

[43] Vecchio GC Del, Giordani L, Santis A De, Mattia D De. Dyserythropoietic anemia and thrombocytopenia due to a novel mutation in {GATA}-1. Acta Haematol. 2005;114: 113–116. doi: 10.1159/000086586 16103636

[44] Mehaffey MG, Newton AL, Gandhi MJ, Crossley M, Drachman JG. X-linked thrombocytopenia caused by a novel mutation of {GATA}-1. Blood. 2001;98: 2681–2688. doi: 10.1182/blood.v98.9.2681 11675338

[45] Freson K, Devriendt K, Matthijs G, Hoof A Van, Vos R De, Thys C, et al. Platelet characteristics in patients with {X}-linked macrothrombocytopenia because of a novel {GATA1} mutation. Blood. 2001;98: 85–92. doi: 10.1182/blood.v98.1.85 11418466

[46] Freson K, Matthijs G, Thys C, Mariën P, Hoylaerts MF, Vermylen J, et al. Different substitutions at residue {D218} of the {X}-linked transcription factor {GATA1} lead to altered clinical severity of macrothrombocytopenia and anemia and are associated with variable skewed {X} inactivation. Hum Mol Genet. 2002;11: 147–152. doi: 10.1093/hmg/11.2.147 11809723

[47] Raskind WH, Niakan KK, Wolff J, Matsushita M, Vaughan T, Stamatoyannopoulos G, et al. Mapping of a syndrome of {X}-linked thrombocytopenia with thalassemia to band {Xp11}-12: further evidence of genetic heterogeneity of {X}-linked thrombocytopenia. Blood. 2000;95: 2262–2268. doi: 10.1182/BLOOD.V95.7.2262 10733494

[48] Hollingsworth SA, Karplus PA. A fresh look at the {Ramachandran} plot and the occurrence of standard structures in proteins. Biomol Concepts. 2010;1: 271–283. doi: 10.1515/BMC.2010.022 21436958 PMC3061398

[49] Mackay JP, Kowalski K, Fox AH, Czolij R, King GF, Crossley M. Involvement of the {N}-finger in the self-association of {GATA}-1. J Biol Chem. 1998;273: 30560–30567. doi: 10.1074/jbc.273.46.30560 9804826

[50] Quax TEF, Claassens NJ, Söll D, Oost J van der. Codon {Bias} as a {Means} to {Fine}-{Tune} {Gene} {Expression}. Mol Cell. 2015;59: 149–161. doi: 10.1016/j.molcel.2015.05.035 26186290 PMC4794256

