# Supplementary figures and images for: The polymorphic landscape analysis of GATA1 exons uncovered the genetic variants associated with higher thrombocytopenia in dengue patients
Source: PLoS Negl Trop Dis. 2022 Jun 30;16(6):e0010537. doi: 10.1371/journal.pntd.0010537 (PMC9278737; doi:10.1371/journal.pntd.0010537)

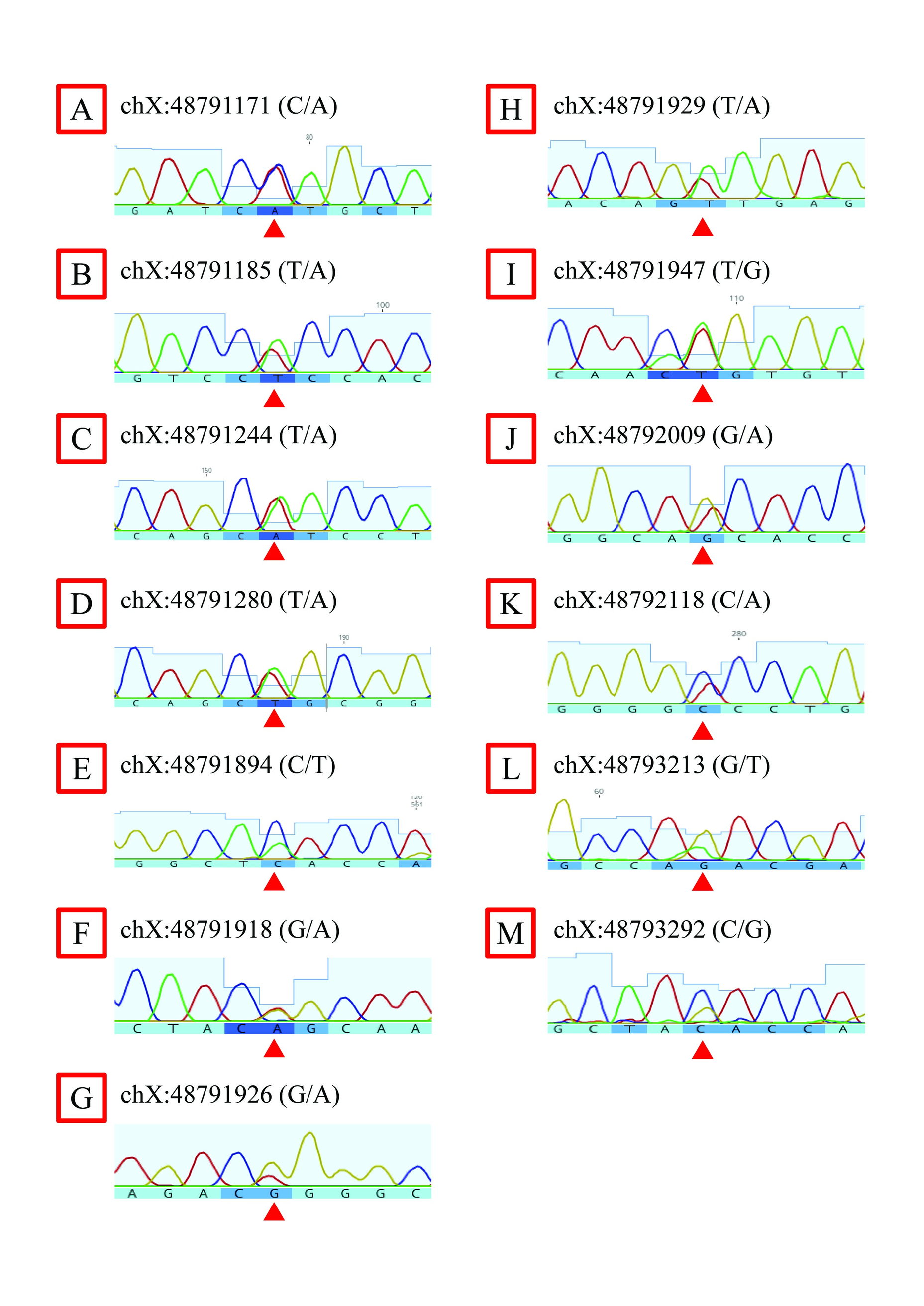

Supplement: S1 Fig — Representing chromatograms obtained through Sanger sequencing of the entire exonic regions of GATA1 gene demonstrating genotypic variations. It has been recognized that all the frequencies represented the heterozygous genotype. (TIF) [file pntd.0010537.s001.tif]
