# Supplementary material for: The polymorphic landscape analysis of GATA1 exons uncovered the genetic variants associated with higher thrombocytopenia in dengue patients
Source: PLoS Negl Trop Dis. 2022 Jun 30;16(6):e0010537. doi: 10.1371/journal.pntd.0010537 (PMC9278737; doi:10.1371/journal.pntd.0010537)
Supplement: S1 Table — (DOCX) [file pntd.0010537.s002.docx]

**Supplementary Table 1.** List of primers designed using Primer3 web based tool to amplify the exonic regions of GATA1 gene.

| Primer set | Primer sequences | | Amplified segments | Amplicon size |
| --- | --- | --- | --- | --- |
|  | Forward | Reverse |  |  |
| 1 | FP-5′-TGTGTCTGAG GACCCCTTCT-3′ | RP-5’-CAATGCCAA GACAGCCACT-3′ | GATA1-  Exon2 | 300bp |
| 2 | FP-5′-TTTGCCTCTTCTT TCCTCCA-3′ | RP 5′-AGGTGAAGC AGGTCTGTGGTT-3′ | GATA1-  Exon3_Exon4 | 690bp |
| 3 | FP-5′- CTTACCCCCA CTTCCACATC-3′ | RP 5′- CAGTGTGGC ATGAAGACAGG-3′ | GATA1-  Exon5 | 239bp |
| 4 | FP-5′- AGTGGGGTA GAGAGGGTGTC-3′ | RP-5’- CATGGTCAC ACATTGCAGCC-3′ | GATA1-  Exon6 | 705bp |
