# Supplementary material for: The polymorphic landscape analysis of GATA1 exons uncovered the genetic variants associated with higher thrombocytopenia in dengue patients
Source: PLoS Negl Trop Dis. 2022 Jun 30;16(6):e0010537. doi: 10.1371/journal.pntd.0010537 (PMC9278737; doi:10.1371/journal.pntd.0010537)
Supplement: S2 Table — (DOCX) [file pntd.0010537.s003.docx]

**Supplementary Table 2.** Optimum conditions applied to perform polymerase chain reaction to amplify targeted regions of GATA1.

| Primer Set | Tm  ( °C) | PCR conditions | | | | |
| --- | --- | --- | --- | --- | --- | --- |
|  |  | 95°C  use | Thermal cycle (40 cycles) | | | 4°C  second |
|  |  |  | 95°C  (seconds) | Tm (seconds) | 72°C  (seconds) |  |
| 1 | 60 | 5 | 30 | 50 | 40 | Hold |
| 2 | 55 | 5 | 30 | 50 | 40 | Hold |
| 3 | 60 | 5 | 30 | 50 | 40 | Hold |
| 4 | 64 | 5 | 30 | 45 | 40 | Hold |
