# Supplementary material for: The polymorphic landscape analysis of GATA1 exons uncovered the genetic variants associated with higher thrombocytopenia in dengue patients
Source: PLoS Negl Trop Dis. 2022 Jun 30;16(6):e0010537. doi: 10.1371/journal.pntd.0010537 (PMC9278737; doi:10.1371/journal.pntd.0010537)
Supplement: S3 Table — (DOCX) [file pntd.0010537.s004.docx]

**Supplementary Table 3.** Prediction of association between GATA1 nonsynonymous mutations and along with their effects and on the stability of the protein.

| **Protein Change** | **PhD SNP** | **SNPs&GO** | **PANTHER-PSEP** | **I-Mutant 2.0 Stability** |
| --- | --- | --- | --- | --- |
| **P21H** | Neutral | Neutral | Possibly Damaging | Decrease |
| **S26T** | Neutral | Neutral | Probably Benign | Decrease |
| **S91L** | Disease | Neutral | Probably benign | Increase |
| **G99S** | Disease | Neutral | Possibly Damaging | Decrease |
| **S129N** | Neutral | Neutral | Probably benign | Increase |
| **Q262H** | Neutral | Neutral | Possibly Damaging | Decrease |
| **H289D** | Disease | Disease | Possibly Damaging | Decrease |
