# Supplementary material for: The polymorphic landscape analysis of GATA1 exons uncovered the genetic variants associated with higher thrombocytopenia in dengue patients
Source: PLoS Negl Trop Dis. 2022 Jun 30;16(6):e0010537. doi: 10.1371/journal.pntd.0010537 (PMC9278737; doi:10.1371/journal.pntd.0010537)
Supplement: S4 Table — (DOCX) [file pntd.0010537.s005.docx]

**Supplementary Table 4.** Association of mean platelet counts with the number of variants harbored by the patients before and after adjusting the confounding factors in female dengue patients.

| **Number of mutations harbored by the participants** | **Number of individuals** | ***Difference (95 % CI)** | **p-value** | ***Difference (95 %CI) ^a+d^** | **p-value^a+d^** |
| --- | --- | --- | --- | --- | --- |
| Zero (no mutations) | 93 |  |  |  |  |
| One | 8 | 18.355(-10.28 to 46.99) | 0.207 | 19.4741(-9.11 to 48.06) | 0.18 |
| Two | 11 | -12.918(-37.7 to 11.87) | 0.304 | -11.4843(-36.26 to 13.29) | 0.36 |
| Three | 2 | -27.145(-82.7 to 28.41) | 0.335 | -23.3963(-79.07 to 32.38) | 0.407 |
| Four | 1 | -3.645(-81.79 to 74.5) | 0.927 | -6.8121(-84.81 to 71.18) | 0.863 |
